# The UK clinical aptitude test and clinical course performance at Nottingham: a prospective cohort study

**DOI:** 10.1186/1472-6920-13-32

**Published:** 2013-02-26

**Authors:** Janet Yates, David James

**Affiliations:** 1 Medical Education Unit, B94 Medical School, Queen’s Medical Centre, Nottingham, NG7 2UH, UK

## Abstract

**Background:**

The UK Clinical Aptitude Test (UKCAT) was introduced in 2006 as an additional tool for the selection of medical students. It tests mental ability in four distinct domains (Verbal Reasoning, Quantitative Reasoning, Abstract Reasoning, and Decision Analysis), and the results are available to students and admission panels in advance of the selection process. Our first study showed little evidence of any predictive validity for performance in the first two years of the Nottingham undergraduate course.

The study objective was to determine whether the UKCAT scores had any predictive value for the later parts of the course, largely delivered via clinical placements.

**Methods:**

Students entering the course in 2007 and who had taken the UKCAT were asked for permission to use their anonymised data in research. The UKCAT scores were incorporated into a database with routine pre-admission socio-demographics and subsequent course performance data. Correlation analysis was followed by hierarchical multivariate linear regression.

**Results:**

The original study group comprised 204/254 (80%) of the full entry cohort. With attrition over the five years of the course this fell to 185 (73%) by Year 5. The Verbal Reasoning score and the UKCAT Total score both demonstrated some univariate correlations with clinical knowledge marks, and slightly less with clinical skills. No parts of the UKCAT proved to be an independent predictor of clinical course marks, whereas prior attainment was a highly significant predictor (p <0.001).

**Conclusions:**

This study of one cohort of Nottingham medical students showed that UKCAT scores at admission did not independently predict subsequent performance on the course. Whilst the test adds another dimension to the selection process, its fairness and validity in selecting promising students remains unproven, and requires wider investigation and debate by other schools.

## Background

The UK Clinical Aptitude Test (UKCAT) has been used since 2006 as an adjunct for the selection of medical and dental students [1]. The need for an additional admissions test has been for three reasons. Firstly, grade inflation over the past decade has made it difficult to distinguish between candidates who all achieve top marks in their final school examinations. Secondly, pupils from state schools in deprived areas, and from some ethnic minority groups, may be disadvantaged, and so there is a need to ‘widen access’ in socio-demographic terms. Finally, there are desirable personal qualities, such as motivation and empathy, required for later professional success, which are difficult to assess. All these factors have been discussed widely elsewhere, and the current literature and evidence has been summarised in a recent Consensus statement [2].

The UKCAT is a test of general intellectual ability rather than factual knowledge, and therefore aims to address the dual problems of grade inflation and widening access. However, UK medical schools use a wide variety of selection procedures [3] and are free to choose whether to include the UKCAT and how they use it. A recent review of the use of the test by all participating medical schools showed that some 7/23 (30%) were using it in some manner to rank or group applicants during selection in 2006, and this proportion had risen to 12/26 (46%) by 2009 [4]. Adam et al. also identified three other broad strategies in which the UKCAT is being used: for borderline decisions on a small number of candidates who were otherwise indistinguishable; as a threshold at a key stage in the selection process, usually after consideration of academic or other criteria; and as a means of ‘rescuing’ candidates by compensating for poorer performance in other parts of the assessment, again affecting a fairly small number of applicants. Some schools used more than one method or have changed their procedure over the ensuing years. It may therefore be difficult to evaluate the UKCAT’s broader contribution to admissions and its subsequent relationship to performance. Beyond the admissions stage, there needs to be some test of long-term validity in terms of students’ success at medical school. Although academic excellence is not the only requirement for a ‘good doctor’, past and current evidence suggests that it is a key predictor of success at medical school [5,6] and beyond [7]. Similar results have been shown in other countries [8-10].

At Nottingham we started to use the UKCAT as a contributory score in our admissions process in 2006 (course entry in 2007). Applicants are scored for their GCSE passes, (A* = 2, A = 1, maximum 24 points), online questionnaire responses (electronic scoring, maximum 29 points), Personal Statement (maximum 12 points), and UKCAT results (scaled to a maximum of 36 points). [11] The UKCAT score therefore represents up to 36% in the scoring system.

We have already shown little correlation between UKCAT scores and course performance during the first two years [11]. This short paper reviews the onward progress of the same cohort and asks whether the UKCAT might predict performance in the later parts of the course.

## Methods

### The study group

The study group comprised all course entrants in 2007 who had taken the UKCAT as part of their admissions procedure and had given consent for their data to be used. We collected routinely-provided demographics (age, sex, domicile, ethnicity and last school type) and each candidate’s UKCAT scores, both as the sub-scores in Verbal Reasoning, Quantitative Reasoning, Abstract Reasoning and Decision Analysis, and the Total score.

We did not utilise the students’ A-level tariff scores because our earlier analysis had shown that these data would have had little discriminatory ability. A large majority (154/193; 80%) of students with A-level data had uniform ‘A’ grades (average tariff = 120), and only two of the remaining 39 had an average tariff score below 110 [11].

### The undergraduate course

The 5-year undergraduate course consists of three stages: two years of largely pre-clinical study, predominantly basic and clinical sciences with summative written exams, plus skills assessment (see Yates & James 2010 for details) [11]; a six-month ‘Honours’ course, comprising an individual research project with thesis and viva, plus some taught courses with written exams; and the clinically-based course, which has three phases over two and a half years. These are:

•Clinical Phase 1 (CP1), duration 6 months, covering Introductory Medicine & Surgery

•Clinical Phase 2 (CP2), duration 12 months, including Obstetrics & Gynaecology, Child Health, Health Care of the Elderly, Psychiatry, and Specials, ie Otorhinolaryngology, Dermatology and Ophthalmology.

•Clinical Phase 3 (CP3), the final year of Advanced Clinical Experience, including Medicine, Surgery, Musculo-skeletal Medicine and General Practice

The overall course assessments utilised in this paper are:

•Part I (weighted average of summative exams and skills assessments in Years 1 and 2)

•Part II (weighted average for Year 3 research project and taught courses)

•Parts I & II weighted average (the entire ‘pre-clinical’ course)

•Weighted CP1 knowledge and skills. The skills examination in this cohort was an OSLER (Objective Structured Long-case Examination and Report), although this has now been replaced by an OSCE (Objective Structured Clinical Examination).

•CP2 weighted average knowledge and skills. All clinical attachments have knowledge exams but there is no OSCE for Specials or for Health Care of the Elderly.

•CP3 weighted average knowledge and skills.

All parts of the course are modular and weighted to generate the required number of credits for course completion. The course also includes other modules, eg student-selected options, which must be passed but are not included in the main assessment scheme.

### Statistical analysis

Examination marks were collected for key stages of the course as shown, collated in Access, and transferred to SPSS v17 for analysis. All continuous data were checked with the 1-sample K-S statistic and found to be normally distributed, with the slight exception of a ‘spike’ in the UKCAT Quantitative Reasoning data. Correlation matrices therefore used Pearson’s correlation coefficient (r) to examine univariate relationships between the UKCAT scores and exam marks. Hierarchical multivariate linear regression was used to determine independent predictors of performance at each stage of the clinical course, by entering variables in three blocks:

1 Socio-demographics (sex, as male = 1, female = 0; ethnicity, as White = 1, non-White = 0; domicile, as Home = 1, EU or overseas = 0; and last school, as selective = 1, state = 0)

2 UKCAT score, either as septe sub-scores or the total

3 Previous course performance as a sequential predictor, ie Parts I & II to predict CP1, adding CP1 to predict CP2, and CP2 to predict CP3.

The outcome variables were the knowledge, skills or combined mark in each clinical stage.

### Ethical approval

As stated above, the students had given written consent for use of their UKCAT scores. Further formal ethical approval was not required by the University of Nottingham Medical School Research Ethics Committee for this analysis of anonymised, routinely-collected data.

## Results

Within the initial cohort of 254 students, 204 (80%) had taken the UKCAT and consented for their data to be used. Attrition and course delay reduced this number to 196 (77%) in CP1, 187 (74%) in CP2 and 185 (73%) in CP3.

Comparison between the study and non-study groups showed no significant differences in socio-demographics, and these data are presented in our previous paper [11].

### Correlations between the UKCAT and course progress

Table 1 shows the correlation matrix between UKCAT scores, with significant but small correlations between most sub-scores. Table 2 shows the correlation matrix for marks across the course; clearly there are highly significant relationships throughout (p < 0.001 in all cases), with the strongest (r >0.6) being between the knowledge-based components. This observation is the basis for the inclusion of prior performance in the hierarchical multivariate regressions.

**Table 1:** Correlations between UKCAT sub-section and total scores

		**UKCAT Verbal reasoning**	**UKCAT Quantitative reasoning**	**UKCAT Abstract reasoning †**	**UKCAT Decision analysis**	**UKCAT Total score**
UKCAT Verbal Reasoning	Pearson r	1				
	Sig. (2-tailed)					
	N	204				
UKCAT Quantitative Reasoning	Pearson r	.221**	1			
	Sig. (2-tailed)	0.002				
	N	204	204			
UKCAT Abstract Reasoning	Pearson r	0.116	.199**	1		
	Sig. (2-tailed)	0.100	0.004			
	N	203	203	203		
UKCAT Decision Analysis	Pearson r	.157*	.190**	.264**	1	
	Sig. (2-tailed)	0.025	0.007	<0.001		
	N	204	204	203	204	
UKCAT Total score	Pearson r	.557**	.546**	.625**	.720**	1
	Sig. (2-tailed)	<0.001	<0.001	<0.001	<0.001	
	N	204	204	203	204	204

**. Correlation is significant at the 0.01 level (2-tailed).

*. Correlation is significant at the 0.05 level (2-tailed).

† the Abstract Reasoning score was missing for one student.

**Table 2:** Correlations between clinical course phases

		**Average Parts I & II**	**CP1 knowledge**	**CP1 skills**	**CP1 average**	**CP2 knowledge**	**CP2 skills**	**CP2 average**	**CP3 knowledge**	**CP3 skills**	**CP3 average**
Average Parts I & II	Pearson r	1									
	Sig. (2-tailed)										
	N	194									
CP1 knowledge	Pearson r	.609**	1								
	Sig. (2-tailed)	<0.001									
	N	194	196								
CP1 skills †	Pearson r	.321**	.426**	1							
	Sig. (2-tailed)	<0.001	<0.001								
	N	194	196	196							
CP1 average	Pearson r	.522**	.793**	.888**	1						
	Sig. (2-tailed)	<0.001	<0.001	<0.001							
	N	194	196	196	196						
CP2 knowledge	Pearson r	.666**	.741**	.452**	.677**	1					
	Sig. (2-tailed)	<0.001	<0.001	<0.001	<0.001						
	N	187	187	187	187	187					
CP2 skills ‡	Pearson r	.515**	.471**	.414**	.515**	.602**	1				
	Sig. (2-tailed)	<0.001	<0.001	<0.001	<0.001	<0.001					
	N	187	187	187	187	187	187				
CP2 average ‡	Pearson r	.662**	.681**	.485**	.669**	.901**	.888**	1			
	Sig. (2-tailed)	<0.001	<0.001	<0.001	<0.001	<0.001	<0.001				
	N	187	187	187	187	187	187	187			
CP3 knowledge	Pearson r	.618**	.669**	.400**	.610**	.783**	.503**	.730**	1		
	Sig. (2-tailed)	<0.001	<0.001	<0.001	<0.001	<0.001	<0.001	<0.001			
	N	185	185	185	185	184	184	184	185		
CP3 skills mark	Pearson r	.430**	.382**	.263**	.372**	.514**	.468**	.553**	.511**	1	
	Sig. (2-tailed)	<0.001	<0.001	<0.001	<0.001	<0.001	<0.001	<0.001	<0.001		
	N	185	185	185	185	184	184	184	185	185	
CP3 average	Pearson r	.607**	.613**	.386**	.572**	.754**	.559**	.743**	.895**	.838**	1
	Sig. (2-tailed)	<0.001	<0.001	<0.001	<0.001	<0.001	<0.001	<0.001	<0.001	<0.001	
	N	185	185	185	185	184	184	184	185	185	185

† in CP1 the skills examination was an OSLER (Objective Structures Long-case Examination and Report).

‡ CP2 skills excluded Health Care of the Elderly.

In view of our previous data, which showed few associations between the UKCAT and the Theme marks from first two years of the course, we re-checked the correlations between the UKCAT and the weighted average of Parts I (weighted Theme averages over the two years) & Part II (weighted average from the 6-month Honours course). There was a minimal correlation with Verbal Reasoning (Pearson r = 0.181, p = 0.011), but none at all with the other sub-scores or the total score (data not shown).

Table 3 shows the correlations between the UKCAT and the clinical phases. The Verbal Reasoning score was the only sub-score to correlate significantly at all stages apart from CP3 skills. Quantitative Reasoning correlated with CP3 knowledge, and weakly with CP1 knowledge and CP3 average. None of the other sub-scores showed any correlations. The total UKCAT score correlated with knowledge but not skills in CP1 and CP3, whereas in CP2 the correlation was higher with skills than with knowledge. In all cases the correlation was relatively weak, r < 0.3.

**Table 3:** Correlations between UKCAT scores and clinical course marks

		**UKCAT Verbal reasoning**	**UKCAT Quantitative reasoning**	**UKCAT Abstract reasoning †**	**UKCAT Decision analysis**	**UKCAT Total score**
CP1 knowledge	Pearson r	.215**	.173*	0.046	0.078	.192**
	Sig. (2-tailed)	0.003	0.015	0.523	0.279	0.007
	N	196	196	195	196	196
CP1 skills	Pearson r	.188**	0.005	0.000	0.049	0.115
	Sig. (2-tailed)	0.008	0.945	0.998	0.494	0.108
	N	196	196	195	196	196
CP1 average	Pearson r	.237**	0.087	0.020	0.070	.173*
	Sig. (2-tailed)	0.001	0.225	0.776	0.327	0.015
	N	196	196	195	196	196
CP2 knowledge	Pearson r	.266**	0.125	0.005	0.086	.176*
	Sig. (2-tailed)	<0.001	0.089	0.947	0.244	0.016
	N	187	187	186	187	187
CP2 skills	Pearson r	.224**	0.126	0.130	0.100	.259**
	Sig. (2-tailed)	0.002	0.086	0.077	0.172	<0.001
	N	187	187	186	187	187
CP2 weighted average	Pearson r	.275**	0.14	0.072	0.104	.242**
	Sig. (2-tailed)	<0.001	0.056	0.326	0.158	0.001
	N	187	187	186	187	187
CP3 knowledge	Pearson r	.255**	.203**	0.004	0.073	.205**
	Sig. (2-tailed)	<0.001	0.006	0.962	0.323	0.005
	N	185	185	184	185	185
CP3 skills	Pearson r	0.144	0.110	0.053	0.020	0.116
	Sig. (2-tailed)	0.050	0.135	0.471	0.791	0.114
	N	185	185	184	185	185
CP3 average	Pearson r	.237**	.183*	0.031	0.060	.193**
	Sig. (2-tailed)	0.001	0.012	0.675	0.414	0.009
	N	185	185	184	185	185

**. Correlation is significant at the 0.01 level (2-tailed).

*. Correlation is significant at the 0.05 level (2-tailed).

† the Abstract Reasoning score was missing for one student.

### Multivariate hierarchical linear regression

A series of regression equations were run with the outcome variables of skills, knowledge or the weighted average at each stage of the clinical course. Only the Verbal and Quantitative Reasoning sub-scores were included as explanatory variables in block 2, since the others had shown no univariate effects. Tables 4 and 5 summarise the statistically significant results for the knowledge and skills components respectively (the full data, including regressions for the weighted averages, are shown in Additional file 1). It is evident that the socio-demographic variables contributed little variance to the models, but had some sustained effects, White ethnicity being a modest positive predictor of CP1 knowledge and CP1 and CP3 skills, and male sex a negative predictor of CP2 knowledge. The two UKCAT scores, and particularly Verbal Reasoning, had some effects in the second regression blocks for knowledge, significantly so for CP2 knowledge. However, the addition of previous performance added substantially to the variance in all models, particularly for knowledge, and emerged as the strongest positive predictor, removing all influence of the UKCAT. The overall average from the early parts of the course (Parts I & II) remained a strong predictor throughout the clinical phases, apart from CP3 knowledge.

**Table 4:** Summary of independent predictors of clinical knowledge (hierarchical linear regression)

**Outcome variable †**	**Block**	**Independent predictors**	**Beta**	**t**	**p**	**R**^**2**^
CP1 knowledge	1	White ethnicity	0.290	3.636	<0.001	0.102
		Selective schooling	−0.157	−2.139	0.034	
	2	White ethnicity	0.255	3.225	0.002	0.167
		Selective schooling	−0.169	−2.363	0.019	
		Verbal Reasoning	0.177	2.381	0.018	
		Quantitative Reasoning	0.166	2.256	0.025	
	3	White ethnicity	0.220	3.334	0.001	0.425
		Parts I & II weighted average	0.529	8.701	<0.001	
CP2 knowledge	1	Male sex	−0.180	−2.396	0.018	0.091
		White ethnicity	0.213	2.604	0.010	
	2	Male sex	−0.225	−3.007	0.003	0.165
		White ethnicity	0.162	2.007	0.046	
		Verbal Reasoning	0.233	3.069	0.003	
	3	Male sex	−0.161	−3.271	0.001	0.647
		Parts I & II weighted average	0.334	5.725	<0.001	
		CP1 knowledge	0.512	8.319	<0.001	
CP3 knowledge	1	(none)				0.041
	2	Verbal Reasoning	0.207	2.669	0.008	0.128
		Quantitative Reasoning	0.191	2.489	0.014	
	3	CP1 knowledge	0.155	2.210	0.029	0.671
		CP2 knowledge	0.609	8.039	<0.001	

* Factors with p > 0.005 are considered not significant (Bonferroni correction for multiple comparisons).

† CP1, CP2, CP3 = successive phases in Clinical Practice.

**Table 5:** Summary of independent predictors of clinical skills (hierarchical linear regression)

**Outcome variable †**	**Block**	**Independent predictors**	**Beta**	**t**	**p**	**R**^**2**^
CP1 skills	1	Home domicile	−0.184	−2.285	0.024	0.086
		White ethnicity	0.293	3.640	<0.001	
	2	Home domicile	−0.197	−2.454	0.015	0.104
		White ethnicity	0.263	3.205	0.002	
	3	Home domicile	−0.167	−2.141	0.034	0.172
		White ethnicity	0.245	3.092	0.002	
		Parts I & II weighted average	0.272	3.737	<0.001	
CP2 skills	1	White ethnicity	0.191	2.314	0.022	0.079
	2	Male sex	−0.187	−2.428	0.016	0.119
	3	Male sex	−0.140	−2.167	0.022	0.389
		Parts I & II weighted average	0.427	6.367	<0.001	
		CP1 skills	0.235	3.459	0.001	
CP3 skills	1	White ethnicity	0.269	3.252	0.001	0.094
	2	Male sex	−0.158	−2.036	0.043	0.117
		White ethnicity	0.264	3.148	0.002	
	3	White ethnicity	0.226	2.962	0.004	0.317
		Parts I & II weighted average	0.280	3.574	<0.001	
		CP2 skills	0.265	3.245	0.001	

* Factors with p > 0.005 are considered not significant (Bonferroni correction for multiple comparisons).

† CP1, CP2, CP3 = successive phases in Clinical Practice.

When the regressions were run using the UKCAT total score instead of the sub-scores, the effects were almost identical to those with the Verbal Reasoning component, with minor differences in actual values but no change in significant predictors (data shown in Additional file 2).

### UKCAT scores and course completion

The database was examined for students who had not graduated on time in 2012. In the entire cohort, there were 28 non-graduates, although 5 of these were still on the course for valid reasons (such as time out for completion of higher degrees) so were counted as ‘successful’ students. The remaining 23 had either left prematurely or had suffered course disruption. A smaller proportion of the study group ‘failed to succeed’, compared to the non-study group (15/204, 7% vs 8/50, 16%), but this was not statistically significant.

Within the study group, the UKCAT scores of the successful and non-successful students were compared. Although the successful group had marginally higher scores (medians 10 or to 20 points higher for each sub-score, and median total score 2550 compared to 2480), these differences were not significant (Mann-Whitney U tests).

### UKCAT scores of applicants and entrants

As stated, Nottingham uses the UKCAT score within the selection process and therefore those students who are accepted are likely to have a different range of scores from those rejected. We compared the UKCAT scores for 208 students awarded places in August 2007 with 1302 students who were rejected. The total score for those accepted was higher (mean 2552 ± 184, compared to 2448 ± 252, p < 0.001). However, the accepted group still had a wide range of scores (2080 to 3020), although less wide than those rejected (1570 to 3190).

## Discussion

This prospective study of one cohort suggests that the UKCAT has very little predictive value for academic performance on the clinical placement phases of the course. Verbal Reasoning showed modest univariate correlation with all clinical course marks scores, with the exception of CP3 skills, but these effects were overwhelmed in regression by the influence of prior course performance. The UKCAT Total score showed similar, but weaker, effects. Students who failed to complete the course on time had lower UKCAT scores, but not statistically so.

The generalisability of these data is limited, not only by the study sample – 80% of one cohort at one medical school – but by the fact that the UKCAT had already been used during the selection process. At the time, the UKCAT score had been scaled to contribute approximately a third of the combined score used to rank candidates [11], and therefore the students subsequently admitted may have had a different range of UKCAT scores than might have otherwise been the case. However, the fact that the UKCAT had already been used to help select these students should not preclude an onwards association with progress, because there is still a wide range of scores in the selected candidates. A-levels have always been used in selection, and have been shown in earlier studies to predict later performance [5,7], but have been considered devalued as a reliable discriminator over recent years through grade inflation and inequitable education. The UKCAT has been developed partly to substitute for A-levels, by picking out students with good intellectual ability [12,13]. It might therefore be expected to select students who will do well on the course, yet it does not appear to be an independent predictor of academic progress on the Nottingham course. However, other intellectual aptitude tests have been shown previously not to predict long-term progress in medicine at other institutions [6]. This finding is borne out by evaluation of another broadly similar test used in Australia and New Zealand, the Undergraduate Medicine and Health Sciences Admission Test (UMAT), which has also shown little predictive ability [9,14]. A study from two Scottish universities has suggested that the UKCAT selects a different profile of student to conventional means of assessment [15]. This leads to questions of how exactly it is working, how it should be used, and whether it is adding to the validity and fairness of selection processes in the longer term. These concerns have also been raised by students who are obliged to sit the test, often at some expense [16-18].

The study excluded 20% of the cohort who did not take the UKCAT or did not give consent for their data to be used. Although this group were similar in socio-demographic profiles, they might not have made equivalent academic progress, so could potentially have affected the results. The additional 19 students who failed to complete on time had marginally lower UKCAT scores. Had they remained in the study, but shown poorer course performance, their data might have increased the correlation between UKCAT and the course marks. However, it is unlikely to have weakened the final regression equation because of the large effect of prior performance.

Currently, the evidence for the predictive validity of the UKCAT, in whichever way it is used, is sparse and equivocal. At two Scottish schools, one of which used the UKCAT score for borderline decisions and the other not at all, there was no relationship with Year 1 outcomes [19]. A study of two cohorts at Newcastle, in which selection procedures had used the UKCAT in different ways in each year, suggested that the UKCAT score was a weak positive predictor of knowledge exams in year 1, but the models did not include prior performance [20]. Hull York medical school did not use the UKCAT within their selection process and have shown some simple correlations between test scores and early course performance, but the authors provided no independent regression analysis [21]. Our own earlier study showed very limited prediction of Themed topics in Years 1 and 2 [11]. Although admissions tests are designed primarily to provide alternative, credible means of selecting students, some longer-term independent associations might have been expected. It is to be hoped that other schools also report on the relationship of the UKCAT to course performance, whilst acknowledging that their other selection procedures, subsequent curricula and examination strategies, will differ from those in Nottingham.

Academic performance is of course not the only criterion for success in medical training and later professional life. Considerable efforts have been made to develop tests for desirable non-cognitive abilities such as interpersonal and communication skills, personality and moral values [22,23]. The UKCAT has previously included a selection of these personality and moral judgement tests (called ‘Section 5’ at the time) but these data were not made available to participating medical schools so remain unevaluated in terms of student progress. However, one medical school has recently reported exploratory data from the same tests taken by their students after admission, and shown some interesting univariate relationships between personality measures and both tutor assessments and conventional examination performance in years 1 and 2 [21]. The UKCAT Board is currently trialling a new set of ‘Situational Judgement Tests’ (SJTs), as are already used for the recruitment of health professionals [24]. Long-term evaluation of these will necessarily take some time and it is hoped that they will eventually contribute an alternative and valid selection tool, offering something that current selection criteria are missing together with some predictive ability.

## Conclusions

This study of one cohort of Nottingham medical students showed that UKCAT scores at admission did not independently predict subsequent performance on the course. Whilst the test adds another dimension to the selection process, its fairness and validity in selecting promising students remains unproven, and requires wider investigation and debate by other schools.

## Competing interests

JY was employed part-time by the UKCAT Board in 2007–08 and DJ was a member of the Board at that time. Neither has any ongoing or current connection with UKCAT and therefore we declare that we have no competing interests.

## Authors’ contributions

Both authors designed the study, contributed to the interpretation of the results and the content of the paper, and approved the final version. JY collected and analysed the data and wrote the first draft of the paper.

## Authors’ information

DJ, Emeritus Professor of Feto-maternal Medicine at Nottingham, was Foundation Director of Medical Education from 2002–2008. JY has been Research Fellow in Medical Education since 2003. Both authors have published widely in the fields of medical student admissions and progress, with a particular focus on the less-successful student.

## Pre-publication history

The pre-publication history for this paper can be accessed here:

http://www.biomedcentral.com/1472-6920/13/32/prepub

## Supplementary Material

Additional file 1 **Full data for Hierarchical Linear Regression of UKCAT sub-scores and clinical course performance.** This file shows the full regression tables for each part of the clinical course (knowledge, skills, and weighted average for CP1, CP2 and CP3), including the UKCAT sub-scores. Click here for file

Additional file 2 **Full data for Hierarchical Linear Regression of UKCAT total score and clinical course performance.** This file shows the full regression tables for each part of the clinical course (knowledge, skills, and weighted average for CP1, CP2 and CP3), including the UKCAT total score. Click here for file
