# Supplementary material for: The UK clinical aptitude test and clinical course performance at Nottingham: a prospective cohort study
Source: BMC Med Educ. 2013 Feb 26;13:32. doi: 10.1186/1472-6920-13-32 (PMC3621812; doi:10.1186/1472-6920-13-32)
Supplement: Additional file 1 — Full data for Hierarchical Linear Regression of UKCAT sub-scores and clinical course performance. This file shows the full regression tables for each part of the clinical course (knowledge, skills, and weighted average for CP1, CP2 and CP3), including the UKCAT sub-scores. [file 1472-6920-13-32-S1.docx]

**Additional File 1**

**Full data for Hierarchical Linear Regression of UKCAT sub-scores and clinical course performance**

**CP1 knowledge**

|  |  |  |  | **Coefficients(a)** | |  |  |  |
| --- | --- | --- | --- | --- | --- | --- | --- | --- |
|  |  | **Unstandardized Coefficients** | | **Standardized Coefficients** | | | **95.0% Confidence Interval for B** | |
| **Model** |  | **B** | **Std. Error** | **Beta** | **t** | **Sig.** | **Lower Bound** | **Upper Bound** |
| 1 | (Constant) | 63.794 | 2.384 |  | 26.758 | 0.000 | 59.088 | 68.500 |
|  | Male sex | 0.050 | 1.348 | 0.003 | 0.037 | 0.970 | -2.609 | 2.710 |
|  | Home student | -3.433 | 2.470 | -0.111 | -1.390 | 0.166 | -8.308 | 1.442 |
|  | White ethnicity | 5.528 | 1.520 | **0.290** | **3.636** | **0.000** | 2.527 | 8.529 |
|  | Selective schooling | -2.953 | 1.380 | **-0.157** | **-2.139** | **0.034** | -5.677 | -0.228 |
| 2 | (Constant) | 35.961 | 8.130 |  | 4.423 | 0.000 | 19.911 | 52.010 |
|  | Male sex | -0.896 | 1.350 | -0.049 | -0.664 | 0.508 | -3.562 | 1.769 |
|  | Home student | -3.946 | 2.405 | -0.127 | -1.641 | 0.103 | -8.694 | 0.802 |
|  | White ethnicity | 4.863 | 1.508 | **0.255** | **3.225** | **0.002** | 1.886 | 7.840 |
|  | Selective schooling | -3.179 | 1.345 | **-0.169** | **-2.363** | **0.019** | -5.834 | -0.523 |
|  | UKCAT Verbal Reasoning | 0.022 | 0.009 | **0.177** | **2.381** | **0.018** | 0.004 | 0.040 |
|  | UKCAT Quantitative Reasoning | 0.024 | 0.011 | **0.166** | **2.256** | **0.025** | 0.003 | 0.046 |
| 3 | (Constant) | -9.452 | 8.553 |  | -1.105 | 0.271 | -26.337 | 7.433 |
|  | Male sex | -0.224 | 1.128 | -0.012 | -0.198 | 0.843 | -2.451 | 2.003 |
|  | Home student | -2.109 | 2.016 | -0.068 | -1.046 | 0.297 | -6.088 | 1.870 |
|  | White ethnicity | 4.198 | 1.259 | **0.220** | **3.334** | **0.001** | 1.712 | 6.683 |
|  | Selective schooling | -1.862 | 1.131 | -0.099 | -1.646 | 0.102 | -4.095 | 0.372 |
|  | UKCAT Verbal Reasoning | 0.010 | 0.008 | 0.080 | 1.275 | 0.204 | -0.005 | 0.025 |
|  | UKCAT Quantitative Reasoning | 0.016 | 0.009 | 0.108 | 1.747 | 0.082 | -0.002 | 0.034 |
|  | Parts I & II Weighted average | 0.969 | 0.111 | **0.529** | **8.701** | **0.000** | 0.750 | 1.189 |
| a. Dependent Variable: CP1 knowledge | |  |  |  |  |  |  |  |

**CP2 knowledge**

|  |  |  |  | **Coefficients(a)** | |  |  |  |
| --- | --- | --- | --- | --- | --- | --- | --- | --- |
|  |  | **Unstandardized Coefficients** | | **Standardized Coefficients** | | | **95.0% Confidence Interval for B** | |
| **Model** |  | **B** | **Std. Error** | **Beta** | **t** | **Sig.** | **Lower Bound** | **Upper Bound** |
| 1 | (Constant) | 66.671 | 1.733 |  | 38.473 | 0.000 | 63.249 | 70.092 |
|  | Male sex | -2.387 | 0.996 | **-0.180** | **-2.396** | **0.018** | -4.355 | -0.420 |
|  | Home student | -1.441 | 1.795 | -0.066 | -0.803 | 0.423 | -4.986 | 2.103 |
|  | White ethnicity | 2.912 | 1.118 | **0.213** | **2.604** | **0.010** | 0.705 | 5.120 |
|  | Selective schooling | -1.598 | 1.020 | -0.118 | -1.567 | 0.119 | -3.611 | 0.415 |
| 2 | (Constant) | 46.167 | 6.090 |  | 7.581 | 0.000 | 34.142 | 58.192 |
|  | Male sex | -2.979 | 0.991 | **-0.225** | **-3.007** | **0.003** | -4.935 | -1.023 |
|  | Home student | -1.912 | 1.739 | -0.087 | -1.100 | 0.273 | -5.345 | 1.521 |
|  | White ethnicity | 2.215 | 1.103 | **0.162** | **2.007** | **0.046** | 0.036 | 4.394 |
|  | Selective schooling | -1.751 | 0.988 | -0.129 | -1.772 | 0.078 | -3.703 | 0.200 |
|  | UKCAT Verbal Reasoning | 0.021 | 0.007 | **0.233** | **3.069** | **0.003** | 0.007 | 0.034 |
|  | UKCAT Quantitative Reasoning | 0.014 | 0.008 | 0.125 | 1.664 | 0.098 | -0.003 | 0.030 |
| 3 | (Constant) | 12.462 | 4.993 |  | 2.496 | 0.014 | 2.602 | 22.323 |
|  | Male sex | -2.129 | 0.651 | **-0.161** | **-3.271** | **0.001** | -3.414 | -0.844 |
|  | Home student | 0.468 | 1.148 | 0.021 | 0.408 | 0.684 | -1.799 | 2.736 |
|  | White ethnicity | 0.099 | 0.747 | 0.007 | 0.133 | 0.894 | -1.375 | 1.574 |
|  | Selective schooling | -0.084 | 0.657 | -0.006 | -0.128 | 0.899 | -1.381 | 1.213 |
|  | UKCAT Verbal Reasoning | 0.005 | 0.005 | 0.061 | 1.187 | 0.237 | -0.004 | 0.014 |
|  | UKCAT Quantitative Reasoning | 0.002 | 0.005 | 0.023 | 0.464 | 0.643 | -0.008 | 0.013 |
|  | Parts I & II Weighted average | 0.439 | 0.077 | **0.334** | **5.725** | **0.000** | 0.287 | 0.590 |
|  | CP1 knowledge | 0.368 | 0.044 | **0.512** | **8.319** | **0.000** | 0.280 | 0.455 |
| a. Dependent Variable: CP2 knowledge | |  |  |  |  |  |  |  |

**CP3 knowledge**

|  |  |  |  | **Coefficients(a)** | |  |  |  |
| --- | --- | --- | --- | --- | --- | --- | --- | --- |
|  |  | **Unstandardized Coefficients** | | **Standardized Coefficients** | | | **95.0% Confidence Interval for B** | |
| **Model** |  | **B** | **Std. Error** | **Beta** | **t** | **Sig.** | **Lower Bound** | **Upper Bound** |
| 1 | (Constant) | 67.137 | 1.729 |  | 38.827 | 0.000 | 63.723 | 70.552 |
|  | Male sex | -0.294 | 1.003 | -0.023 | -0.293 | 0.770 | -2.274 | 1.686 |
|  | Home student | -1.732 | 1.800 | -0.082 | -0.962 | 0.337 | -5.285 | 1.822 |
|  | White ethnicity | 2.241 | 1.136 | 0.168 | 1.972 | 0.050 | -0.002 | 4.485 |
|  | Selective schooling | -1.522 | 1.027 | -0.116 | -1.481 | 0.140 | -3.551 | 0.507 |
| 2 | (Constant) | 43.924 | 6.129 |  | 7.167 | 0.000 | 31.821 | 56.027 |
|  | Male sex | -1.012 | 0.989 | -0.079 | -1.024 | 0.307 | -2.965 | 0.940 |
|  | Home student | -2.223 | 1.737 | -0.105 | -1.280 | 0.203 | -5.654 | 1.208 |
|  | White ethnicity | 1.734 | 1.111 | 0.130 | 1.561 | 0.120 | -0.459 | 3.927 |
|  | Selective schooling | -1.650 | 0.991 | -0.126 | -1.666 | 0.098 | -3.606 | 0.306 |
|  | UKCAT Verbal Reasoning | 0.018 | 0.007 | **0.207** | **2.669** | **0.008** | 0.005 | 0.031 |
|  | UKCAT Quantitative Reasoning | 0.020 | 0.008 | **0.191** | **2.489** | **0.014** | 0.004 | 0.036 |
| 3 | (Constant) | 4.255 | 4.895 |  | 0.869 | 0.386 | -5.412 | 13.923 |
|  | Male sex | 1.000 | 0.638 | 0.078 | 1.567 | 0.119 | -0.260 | 2.259 |
|  | Home student | -0.741 | 1.085 | -0.035 | -0.683 | 0.495 | -2.885 | 1.402 |
|  | White ethnicity | 0.076 | 0.709 | 0.006 | 0.107 | 0.915 | -1.325 | 1.476 |
|  | Selective schooling | -0.079 | 0.625 | -0.006 | -0.126 | 0.900 | -1.313 | 1.156 |
|  | UKCAT Verbal Reasoning | 0.003 | 0.004 | 0.030 | 0.608 | 0.544 | -0.006 | 0.011 |
|  | UKCAT Quantitative Reasoning | 0.010 | 0.005 | 0.092 | 1.912 | 0.058 | 0.000 | 0.020 |
|  | Parts I & II Weighted average | 0.131 | 0.080 | 0.101 | 1.631 | 0.105 | -0.028 | 0.290 |
|  | CP1 knowledge | 0.110 | 0.050 | **0.155** | **2.210** | **0.029** | 0.012 | 0.208 |
|  | CP2 knowledge | 0.605 | 0.075 | **0.609** | **8.039** | **0.000** | 0.457 | 0.754 |
| a. Dependent Variable: CP3 knowledge | |  |  |  |  |  |  |  |

**CP1 skills**

|  |  |  |  | **Coefficients(a)** | |  |  |  |
| --- | --- | --- | --- | --- | --- | --- | --- | --- |
|  |  | **Unstandardized Coefficients** | | **Standardized Coefficients** | | | **95.0% Confidence Interval for B** | |
| **Model** |  | **B** | **Std. Error** | **Beta** | **t** | **Sig.** | **Lower Bound** | **Upper Bound** |
| 1 | (Constant) | 78.301 | 3.259 |  | 24.024 | 0.000 | 71.868 | 84.735 |
|  | Male sex | -0.851 | 1.842 | -0.034 | -0.462 | 0.645 | -4.487 | 2.785 |
|  | Home student | -7.716 | 3.376 | **-0.184** | **-2.285** | **0.024** | -14.380 | -1.051 |
|  | White ethnicity | 7.566 | 2.078 | **0.293** | **3.640** | **0.000** | 3.464 | 11.668 |
|  | Selective schooling | -1.999 | 1.887 | -0.078 | -1.059 | 0.291 | -5.723 | 1.726 |
| 2 | (Constant) | 64.164 | 11.426 |  | 5.616 | 0.000 | 41.609 | 86.719 |
|  | Male sex | -1.074 | 1.898 | -0.043 | -0.566 | 0.572 | -4.820 | 2.672 |
|  | Home student | -8.295 | 3.380 | **-0.197** | **-2.454** | **0.015** | -14.967 | -1.623 |
|  | White ethnicity | 6.793 | 2.119 | **0.263** | **3.205** | **0.002** | 2.609 | 10.976 |
|  | Selective schooling | -2.319 | 1.891 | -0.091 | -1.227 | 0.222 | -6.051 | 1.413 |
|  | UKCAT Verbal Reasoning | 0.023 | 0.013 | 0.137 | 1.770 | 0.079 | -0.003 | 0.048 |
|  | UKCAT Quantitative Reasoning | 0.002 | 0.015 | 0.009 | 0.119 | 0.905 | -0.028 | 0.032 |
| 3 | (Constant) | 32.466 | 13.901 |  | 2.335 | 0.021 | 5.023 | 59.909 |
|  | Male sex | -0.604 | 1.834 | -0.024 | -0.330 | 0.742 | -4.224 | 3.015 |
|  | Home student | -7.013 | 3.276 | **-0.167** | **-2.141** | **0.034** | -13.480 | -0.545 |
|  | White ethnicity | 6.328 | 2.046 | **0.245** | **3.092** | **0.002** | 2.288 | 10.368 |
|  | Selective schooling | -1.400 | 1.839 | -0.055 | -0.761 | 0.447 | -5.030 | 2.230 |
|  | UKCAT Verbal Reasoning | 0.014 | 0.013 | 0.087 | 1.147 | 0.253 | -0.010 | 0.039 |
|  | UKCAT Quantitative Reasoning | -0.004 | 0.015 | -0.021 | -0.283 | 0.778 | -0.033 | 0.025 |
|  | Parts I & II Weighted average | 0.677 | 0.181 | **0.272** | **3.737** | **0.000** | 0.319 | 1.034 |
| a. Dependent Variable: CP1 skills | |  |  |  |  |  |  |  |

**CP2 skills**

|  |  |  |  | **Coefficients(a)** | |  |  |  |
| --- | --- | --- | --- | --- | --- | --- | --- | --- |
|  |  | **Unstandardized Coefficients** | | **Standardized Coefficients** | | | **95.0% Confidence Interval for B** | |
| **Model** |  | **B** | **Std. Error** | **Beta** | **t** | **Sig.** | **Lower Bound** | **Upper Bound** |
| 1 | (Constant) | 65.098 | 1.783 |  | 36.514 | 0.000 | 61.578 | 68.617 |
|  | Male sex | -1.958 | 1.025 | -0.145 | -1.910 | 0.058 | -3.981 | 0.066 |
|  | Home student | 1.036 | 1.847 | 0.046 | 0.561 | 0.576 | -2.611 | 4.682 |
|  | White ethnicity | 2.662 | 1.150 | **0.191** | **2.314** | **0.022** | 0.391 | 4.934 |
|  | Selective schooling | -1.304 | 1.049 | -0.094 | -1.243 | 0.216 | -3.375 | 0.768 |
| 2 | (Constant) | 48.395 | 6.392 |  | 7.571 | 0.000 | 35.774 | 61.017 |
|  | Male sex | -2.525 | 1.040 | **-0.187** | **-2.428** | **0.016** | -4.579 | -0.472 |
|  | Home student | 0.771 | 1.825 | 0.034 | 0.423 | 0.673 | -2.832 | 4.375 |
|  | White ethnicity | 2.259 | 1.158 | 0.162 | 1.950 | 0.053 | -0.028 | 4.546 |
|  | Selective schooling | -1.349 | 1.037 | -0.098 | -1.300 | 0.195 | -3.397 | 0.700 |
|  | UKCAT Verbal Reasoning | 0.012 | 0.007 | 0.135 | 1.732 | 0.085 | -0.002 | 0.026 |
|  | UKCAT Quantitative Reasoning | 0.015 | 0.009 | 0.137 | 1.779 | 0.077 | -0.002 | 0.032 |
| 3 | (Constant) | 13.208 | 6.806 |  | 1.941 | 0.054 | -0.232 | 26.648 |
|  | Male sex | -1.897 | 0.875 | **-0.140** | **-2.167** | **0.032** | -3.625 | -0.168 |
|  | Home student | 2.942 | 1.559 | 0.131 | 1.887 | 0.061 | -0.136 | 6.021 |
|  | White ethnicity | 1.018 | 0.997 | 0.073 | 1.021 | 0.309 | -0.952 | 2.987 |
|  | Selective schooling | -0.320 | 0.878 | -0.023 | -0.365 | 0.716 | -2.055 | 1.414 |
|  | UKCAT Verbal Reasoning | 0.001 | 0.006 | 0.012 | 0.186 | 0.852 | -0.011 | 0.013 |
|  | UKCAT Quantitative Reasoning | 0.011 | 0.007 | 0.102 | 1.569 | 0.119 | -0.003 | 0.026 |
|  | Parts I & II Weighted average | 0.573 | 0.090 | **0.427** | **6.367** | **0.000** | 0.395 | 0.751 |
|  | CP1 skills | 0.128 | 0.037 | **0.235** | **3.459** | **0.001** | 0.055 | 0.201 |
| a. Dependent Variable: CP2 skills | |  |  |  |  |  |  |  |

**CP3 skills**

|  |  |  |  | **Coefficients(a)** | |  |  |  |
| --- | --- | --- | --- | --- | --- | --- | --- | --- |
|  |  | **Unstandardized Coefficients** | | **Standardized Coefficients** | | | **95.0% Confidence Interval for B** | |
| **Model** |  | **B** | **Std. Error** | **Beta** | **t** | **Sig.** | **Lower Bound** | **Upper Bound** |
| 1 | (Constant) | 67.611 | 1.328 |  | 50.916 | 0.000 | 64.989 | 70.233 |
|  | Male sex | -1.238 | 0.770 | -0.122 | -1.608 | 0.110 | -2.759 | 0.282 |
|  | Home student | -0.515 | 1.382 | -0.031 | -0.372 | 0.710 | -3.244 | 2.214 |
|  | White ethnicity | 2.838 | 0.873 | **0.269** | **3.252** | **0.001** | 1.115 | 4.561 |
|  | Selective schooling | -0.972 | 0.789 | -0.094 | -1.232 | 0.220 | -2.530 | 0.586 |
| 2 | (Constant) | 58.409 | 4.873 |  | 11.987 | 0.000 | 48.786 | 68.031 |
|  | Male sex | -1.600 | 0.786 | **-0.158** | **-2.036** | **0.043** | -3.153 | -0.048 |
|  | Home student | -0.585 | 1.381 | -0.035 | -0.424 | 0.672 | -3.313 | 2.142 |
|  | White ethnicity | 2.780 | 0.883 | **0.264** | **3.148** | **0.002** | 1.036 | 4.524 |
|  | Selective schooling | -0.952 | 0.788 | -0.092 | -1.209 | 0.228 | -2.507 | 0.603 |
|  | UKCAT Verbal Reasoning | 0.003 | 0.005 | 0.040 | 0.517 | 0.606 | -0.008 | 0.013 |
|  | UKCAT Quantitative Reasoning | 0.012 | 0.006 | 0.143 | 1.853 | 0.066 | -0.001 | 0.025 |
| 3 | (Constant) | 34.116 | 5.661 |  | 6.026 | 0.000 | 22.935 | 45.297 |
|  | Male sex | -0.898 | 0.710 | -0.088 | -1.265 | 0.208 | -2.301 | 0.504 |
|  | Home student | -0.483 | 1.265 | -0.029 | -0.382 | 0.703 | -2.982 | 2.015 |
|  | White ethnicity | 2.378 | 0.803 | **0.226** | **2.962** | **0.004** | 0.793 | 3.963 |
|  | Selective schooling | -0.238 | 0.709 | -0.023 | -0.336 | 0.738 | -1.638 | 1.162 |
|  | UKCAT Verbal Reasoning | -0.002 | 0.005 | -0.036 | -0.504 | 0.615 | -0.012 | 0.007 |
|  | UKCAT Quantitative Reasoning | 0.007 | 0.006 | 0.079 | 1.137 | 0.257 | -0.005 | 0.018 |
|  | Parts I & II Weighted average | 0.288 | 0.081 | **0.280** | **3.574** | **0.000** | 0.129 | 0.447 |
|  | CP1 skills | -0.002 | 0.030 | -0.004 | -0.060 | 0.952 | -0.062 | 0.058 |
|  | CP2 skills | 0.210 | 0.065 | **0.265** | **3.245** | **0.001** | 0.082 | 0.338 |
| a. Dependent Variable: Average CP3 skills mark | | | |  |  |  |  |  |

**CP1 weighted average**

|  |  |  |  | **Coefficients(a)** | |  |  |  |
| --- | --- | --- | --- | --- | --- | --- | --- | --- |
|  |  | **Unstandardized Coefficients** | | **Standardized Coefficients** | | | **95.0% Confidence Interval for B** | |
| **Model** |  | **B** | **Std. Error** | **Beta** | **t** | **Sig.** | **Lower Bound** | **Upper Bound** |
| 1 | (Constant) | 71.243 | 2.369 |  | 30.067 | 0.000 | 66.566 | 75.919 |
|  | Male sex | -0.400 | 1.339 | -0.022 | -0.299 | 0.766 | -3.043 | 2.243 |
|  | Home student | -5.529 | 2.455 | **-0.177** | **-2.253** | **0.026** | -10.374 | -0.684 |
|  | White ethnicity | 6.575 | 1.511 | **0.343** | **4.352** | **0.000** | 3.593 | 9.557 |
|  | Selective schooling | -2.405 | 1.372 | -0.127 | -1.753 | 0.081 | -5.112 | 0.303 |
| 2 | (Constant) | 50.429 | 8.181 |  | 6.164 | 0.000 | 34.280 | 66.579 |
|  | Male sex | -0.968 | 1.359 | -0.052 | -0.713 | 0.477 | -3.651 | 1.714 |
|  | Home student | -6.085 | 2.420 | **-0.195** | **-2.514** | **0.013** | -10.862 | -1.308 |
|  | White ethnicity | 5.842 | 1.517 | **0.305** | **3.850** | **0.000** | 2.847 | 8.837 |
|  | Selective schooling | -2.685 | 1.354 | -0.142 | -1.983 | 0.049 | -5.357 | -0.013 |
|  | UKCAT Verbal Reasoning | 0.023 | 0.009 | **0.183** | **2.457** | **0.015** | 0.004 | 0.041 |
|  | UKCAT Quantitative Reasoning | 0.012 | 0.011 | 0.085 | 1.150 | 0.252 | -0.009 | 0.034 |
| 3 | (Constant) | 11.572 | 9.125 |  | 1.268 | 0.206 | -6.441 | 29.586 |
|  | Male sex | -0.393 | 1.204 | -0.021 | -0.326 | 0.745 | -2.769 | 1.983 |
|  | Home student | -4.513 | 2.150 | **-0.145** | **-2.099** | **0.037** | -8.759 | -0.268 |
|  | White ethnicity | 5.273 | 1.343 | **0.275** | **3.925** | **0.000** | 2.621 | 7.925 |
|  | Selective schooling | -1.558 | 1.207 | -0.082 | -1.291 | 0.198 | -3.941 | 0.824 |
|  | UKCAT Verbal Reasoning | 0.012 | 0.008 | 0.101 | 1.504 | 0.134 | -0.004 | 0.029 |
|  | UKCAT Quantitative Reasoning | 0.005 | 0.010 | 0.035 | 0.537 | 0.592 | -0.014 | 0.024 |
|  | Parts I & II Weighted average | 0.830 | 0.119 | **0.450** | **6.979** | **0.000** | 0.595 | 1.064 |
| a. Dependent Variable: CP1 average | |  |  |  |  |  |  |  |

**CP2 weighted average**

|  |  |  |  | **Coefficients(a)** | |  |  |  |
| --- | --- | --- | --- | --- | --- | --- | --- | --- |
|  |  | **Unstandardized Coefficients** | | **Standardized Coefficients** | | | **95.0% Confidence Interval for B** | |
| **Model** |  | **B** | **Std. Error** | **Beta** | **t** | **Sig.** | **Lower Bound** | **Upper Bound** |
| 1 | (Constant) | 65.917 | 1.573 |  | 41.915 | 0.000 | 62.812 | 69.022 |
|  | Male sex | -2.182 | 0.904 | **-0.181** | **-2.413** | **0.017** | -3.967 | -0.396 |
|  | Home student | -0.255 | 1.629 | -0.013 | -0.156 | 0.876 | -3.471 | 2.962 |
|  | White ethnicity | 2.793 | 1.015 | **0.224** | **2.752** | **0.007** | 0.789 | 4.796 |
|  | Selective schooling | -1.457 | 0.925 | -0.118 | -1.574 | 0.117 | -3.284 | 0.370 |
| 2 | (Constant) | 47.235 | 5.543 |  | 8.521 | 0.000 | 36.289 | 58.180 |
|  | Male sex | -2.762 | 0.902 | **-0.229** | **-3.062** | **0.003** | -4.542 | -0.981 |
|  | Home student | -0.627 | 1.583 | -0.031 | -0.396 | 0.693 | -3.751 | 2.498 |
|  | White ethnicity | 2.236 | 1.004 | **0.180** | **2.226** | **0.027** | 0.253 | 4.219 |
|  | Selective schooling | -1.558 | 0.900 | -0.126 | -1.732 | 0.085 | -3.335 | 0.218 |
|  | UKCAT Verbal Reasoning | 0.017 | 0.006 | **0.206** | **2.713** | **0.007** | 0.005 | 0.029 |
|  | UKCAT Quantitative Reasoning | 0.014 | 0.007 | 0.145 | 1.935 | 0.055 | 0.000 | 0.029 |
| 3 | (Constant) | 9.973 | 4.799 |  | 2.078 | 0.039 | 0.495 | 19.450 |
|  | Male sex | -2.037 | 0.624 | **-0.169** | **-3.264** | **0.001** | -3.269 | -0.804 |
|  | Home student | 1.973 | 1.111 | 0.098 | 1.775 | 0.078 | -0.222 | 4.167 |
|  | White ethnicity | 0.380 | 0.723 | 0.030 | 0.525 | 0.600 | -1.048 | 1.807 |
|  | Selective schooling | -0.205 | 0.628 | -0.017 | -0.326 | 0.745 | -1.446 | 1.036 |
|  | UKCAT Verbal Reasoning | 0.003 | 0.004 | 0.035 | 0.643 | 0.521 | -0.006 | 0.011 |
|  | UKCAT Quantitative Reasoning | 0.008 | 0.005 | 0.085 | 1.653 | 0.100 | -0.002 | 0.019 |
|  | Parts I & II Weighted average | 0.510 | 0.070 | **0.425** | **7.291** | **0.000** | 0.372 | 0.648 |
|  | CP1 average | 0.261 | 0.040 | **0.401** | **6.498** | **0.000** | 0.182 | 0.341 |
| a. Dependent Variable: CP2 weighted average (excl HCE skills) | | | |  |  |  |  |  |

**CP3 weighted average**

|  |  |  |  | **Coefficients(a)** | |  |  |  |
| --- | --- | --- | --- | --- | --- | --- | --- | --- |
|  |  | **Unstandardized Coefficients** | | **Standardized Coefficients** | | | **95.0% Confidence Interval for B** | |
| **Model** |  | **B** | **Std. Error** | **Beta** | **t** | **Sig.** | **Lower Bound** | **Upper Bound** |
| 1 | (Constant) | 67.670 | 1.317 |  | 51.396 | 0.000 | 65.071 | 70.270 |
|  | Male sex | -0.768 | 0.764 | -0.077 | -1.005 | 0.316 | -2.276 | 0.740 |
|  | Home student | -1.165 | 1.370 | -0.071 | -0.850 | 0.396 | -3.871 | 1.541 |
|  | White ethnicity | 2.559 | 0.865 | **0.247** | **2.958** | **0.004** | 0.851 | 4.268 |
|  | Selective schooling | -1.268 | 0.782 | -0.124 | -1.621 | 0.107 | -2.813 | 0.277 |
| 2 | (Constant) | 51.472 | 4.710 |  | 10.927 | 0.000 | 42.170 | 60.774 |
|  | Male sex | -1.307 | 0.760 | -0.131 | -1.720 | 0.087 | -2.808 | 0.193 |
|  | Home student | -1.446 | 1.335 | -0.088 | -1.083 | 0.280 | -4.083 | 1.190 |
|  | White ethnicity | 2.276 | 0.854 | **0.220** | **2.667** | **0.008** | 0.591 | 3.962 |
|  | Selective schooling | -1.322 | 0.761 | -0.129 | -1.737 | 0.084 | -2.826 | 0.181 |
|  | UKCAT Verbal Reasoning | 0.010 | 0.005 | **0.154** | **2.006** | **0.047** | 0.000 | 0.021 |
|  | UKCAT Quantitative Reasoning | 0.016 | 0.006 | **0.195** | **2.574** | **0.011** | 0.004 | 0.028 |
| 3 | (Constant) | 17.073 | 4.234 |  | 4.033 | 0.000 | 8.712 | 25.435 |
|  | Male sex | 0.085 | 0.548 | 0.009 | 0.155 | 0.877 | -0.997 | 1.168 |
|  | Home student | -0.902 | 0.954 | -0.055 | -0.946 | 0.346 | -2.786 | 0.982 |
|  | White ethnicity | 1.214 | 0.616 | 0.117 | 1.972 | 0.050 | -0.002 | 2.430 |
|  | Selective schooling | -0.178 | 0.538 | -0.017 | -0.330 | 0.742 | -1.239 | 0.884 |
|  | UKCAT Verbal Reasoning | 0.001 | 0.004 | 0.009 | 0.174 | 0.862 | -0.007 | 0.008 |
|  | UKCAT Quantitative Reasoning | 0.008 | 0.004 | 0.100 | 1.883 | 0.061 | 0.000 | 0.017 |
|  | Parts I & II Weighted average | 0.198 | 0.069 | **0.196** | **2.876** | **0.005** | 0.062 | 0.334 |
|  | CP1 average | 0.044 | 0.038 | 0.080 | 1.150 | 0.252 | -0.031 | 0.119 |
|  | CP2 weighted average (excl HCE skills) | 0.455 | 0.068 | **0.526** | **6.659** | **0.000** | 0.320 | 0.590 |
| a. Dependent Variable: CP3 average knowledge & skills | | |  |  |  |  |  |  |
